# Practice, Skill Mix, and Education: The Evolving Role of Pharmacy Technicians in Great Britain

**DOI:** 10.3390/pharmacy8020050

**Published:** 2020-03-26

**Authors:** Melanie Boughen, Tess Fenn

**Affiliations:** 1 School of Pharmacy, University of East Anglia, Norwich NR4 7TJ, UK; 2 European Association of Pharmacy Technicians, 2500 Valby, Denmark; eaptsecretary@outlook.com

**Keywords:** pharmacy technician, education, skill mix, extended roles, patient safety, medicines reconciliation

## Abstract

Pharmacy technicians’ roles are rapidly evolving in Great Britain (GB) as they undertake more extended activities with increased autonomy across the different pharmacy sectors. This paper compares the GB pharmacy regulator initial education and training standards recently introduced (2017) with the qualifications currently used in practice and discusses whether future qualifications will be ‘fit for purpose’. In this context, knowledge, skills, and competence are reviewed to assess whether they will meet the expectations and underpin the evolving pharmacy technician role as integral to healthcare provision. Based on drivers, policy change, and the changing GB healthcare landscape, effectiveness of skill mix is analysed to establish whether this is being optimised to support person-centred pharmacy in response to the challenges and pressures faced within the NHS. On this basis and given there is a limited evidence base, this review has highlighted a need for larger scale research to reassure the pharmacy and wider healthcare professions, and the public, that the evolving pharmacy technician role presents no increased risk to patient safety and contributes significantly to releasing pharmacists time for person-centred clinical activities.

## 1. Introduction

Pharmacy technicians in Great Britain were first accepted onto the General Pharmaceutical Council (GPhC) register, the pharmacy regulator, in 2011 and practice to the same GPhC professional standards as pharmacists [1]. Since registration, they have gained increased recognition for their contribution to the healthcare agenda as their roles, scope of practice, and autonomy increase. This is partly due to the realisation that many ‘traditional’ pharmacists’ roles have become increasingly technical due to the introduction of automation and enhancement of information technology as well as the changing focus of practice to become person/patient-centred. 

As a result, particularly in the hospital sector, with specific training, these extended roles have evolved from traditional pharmacy technician activities such as dispensing and stock management, to final accuracy checking (in the UK this is a nonclinical check for accuracy of prescribed and dispensed medicines, as opposed to the clinical check conducted by a pharmacist for clinical appropriateness for a patient), medicines optimisation skills* (see Table 1), and pharmacy management. All of these roles have previously been traditional pharmacists’ roles. However, although the UK government vision for the community pharmacist role has significantly changed from the supply of medicines to the clinical provision of patient care—and this gives opportunities for pharmacy technician role development—this has been slower to evolve in community pharmacy. Currently, pharmacy legislation relating to the ‘supervision’ of the preparation, assembly, sale, and supply, including dispensing, of medicines from GPhC-registered pharmacies prevents pharmacists from leaving the pharmacy for significant periods of time [2]. Therefore, pharmacy technicians have not been able to assume responsibility for all activity within the dispensary in the same manner as that seen in the hospital setting.

Albeit, at a varying pace across the different sectors and settings, the pharmacy technician landscape is changing, and in addition to the traditional settings of community and hospital, pharmacy technicians are now increasingly located within care homes and GP Practices (doctors surgeries and health centres) performing very much a clinical role as part of medicines optimisation* teams.

In recent years, there has been some growth in the body of literature on extended/advance roles and although these are generally limited to small local studies, they do provide insight into pharmacy technician role development [3,4,5,6,7]. The literature also highlights the positive contribution that pharmacy technicians, with specific training, can make to pharmacy services and patient care, with the general theme that the extended roles release pharmacists for more patient-facing clinical activity and further developing their clinical skills and knowledge to train as nonmedical prescribers. On a more global scale, it should be noted that releasing pharmacists time is not always the reason that pharmacy technicians scope and autonomy has increased—there are countries with rural and remote environments and populations where with little infrastructure and few pharmacists, pharmacy technicians and other support staff operate autonomously out of necessity [8]. 

To better understand the evolving role in GB, in this article we will look at the main drivers for change of the pharmacy technician role, how the role has evolved in response to this, what needs to change to support the transition (education), and finally, how the role may evolve further in the future.

## 2. Drivers and Responding to Change in Great Britain

In pharmacy, as well as all other sectors of healthcare, there never seems a point when the workforce is not under extreme pressure to deliver services. This has led to several NHS ‘White Paper’ publications including The Interim NHS People Plan [10] and the Interim NHS People Plan: the future pharmacy workforce [11], which state the importance of pharmacy involvement in patient and public care and identifying the support that pharmacy technicians can provide across different sectors, practicing to the ‘top of their licence’. The NHS England (NHSE) review on secondary care productivity in NHS Hospitals [12] (commonly known as the Carter Review) and most recently in 2020, the NHS England Update to the GP Contract [13], formally recognise pharmacy technicians alongside pharmacists as part of the skill mix needed to deliver person-centred care. It is through this formal recognition that pharmacy technicians as healthcare professionals can progress further alongside other healthcare professions.

For changes to be successful, understanding skill mix efficiency (ensuring the right people, with the right skills, are in the right place at the right time) and what can be achieved by maximising skill mix is critical. Poorly managed skill mix to just ‘get a job done’ could be counterproductive and a risk to patient safety. McIntosh and Sheppy highlighted that productivity and safety can be enhanced simultaneously by greater use of the skills and experiences of all staff and could enhance outcomes both clinically and economically [14].

Arguably, in the UK, there still remains some confusion as to pharmacy technicians’ scope of practice, role boundaries, and accountability. This is more prevalent in the community sector, where there is often a blurring of pharmacy technician and pharmacy/dispensing assistant roles. One activity that does separate pharmacy technicians from pharmacy assistants is final accuracy checking, and a major training and development-funded initiative was introduced in 2016 by NHS England (Pharmacy Integration Fund) [15]. The intention of this ongoing initiative is to drive the greater use of pharmacists and pharmacy technicians in new, integrated local care models. Part of this initiative is to broaden the skills of pharmacy technicians working in the community sector by funding final accuracy checking—however, as this is still ongoing, no evaluation of its success is available.

In comparison, in the UK, the pharmacy assistant is an essential member of the pharmacy team and assists pharmacists and pharmacy technicians in both community and hospital pharmacy settings. In the secondary care setting, there is more variety and clarity of the role, whereas in community the role generally focuses on stock maintenance and the assembly aspect of the dispensing process but with less demarcation of responsibilities within the pharmacy team. On-the-job training, equivalent to UK level 2, is provided to meet the GPhC education requirements, however the pharmacy assistant is not a registrant.

Another contributing factor in community pharmacy is the use of locum pharmacists who may not be familiar with the team, and therefore be less forthcoming or possibly less confident in delegating tasks when they are the ‘Responsible Pharmacist’ [16]. However, this is not always the reason, and sometimes it is time pressures on management that prevent implementation of skill mix strategy or staff that recognise greater use of extended roles and responsibilities [17] but may not feel empowered to influence any change. Although this is occurring less, it remains a barrier and can restrict flow of patient services. According to West [18], organisational skill mix reviews are key to ascertain what activities need to be carried out, who has the minimum level of skills to undertake them, and if new roles need to be created to fulfil optimisation. Pharmacists spending time on ‘traditional’ roles do not optimise their skills as they do not need to final-accuracy-check prescriptions, manage the day-to-day supervision of staff, or prepare staff rotas—which are technical duties. With effective communication, robust procedures, and clear understanding of boundaries and lines of responsibility, the majority of pharmacy technicians have the knowledge and skills or the potential to undertake these activities. As many pharmacists are managers, another aspect for making skill mix work is recognising the needs of the pharmacist—pharmacists need developed skills in delegation and managing teams, which some see as their own development requirements [19].

With further regard to skill mix, emerging evidence does not suggest that pharmacy technicians are less safe when taking on extended roles. Rather, it suggests that because they are trained for the specific role, they are likely to have fewer competing demands and have been found to have a higher level of accuracy than pharmacists and other healthcare professionals [20,21,22]. However, this evidence is still very limited with small scale studies and wider, larger scale research needs to be undertaken to reassure pharmacists and wider healthcare teams that from these roles there is no worsening risk to patient safety or systems.

## 3. Education

In light of the changing roles of pharmacy technicians, education is pivotal, and a key point of interest is the level of the baseline pharmacy technician qualifications, which vary considerably from country to country.

In 2017, the European Association of Pharmacy Technicians (EAPT) undertook a European-wide survey of education and training programmes [23]. The results highlighted the variation in pharmacy technician education in Europe with levels of study required for practice, licensing, or registration ranging from post-secondary diplomas to bachelor’s degrees. Interestingly, comparison between the level of initial education requirements and the role undertaken by community pharmacy technicians in the EAPT 2017 European Survey [23] shows a correlation between countries with higher level education requirements and pharmacy practice activities. The volume and complexity of the dispensing activities carried out and the application of problem-solving skills are specifically enhanced in Denmark and Portugal, where bachelor’s degrees are in place as the baseline education. Interestingly, comparing the academic credits of these bachelor’s degrees using the European Credit Transfer and Accumulation System (ECTS) shows a further differential between the countries, with Denmark accruing 180 ECTS credits and Portugal 240.

In Great Britain, trainee pharmacy technicians are known as preregistration trainee pharmacy technicians (PTPTs) and undertake their training over a two-year period. Trainees are employed, therefore much of their learning and competence is gained in the workplace as they complete a competence-based qualification. They are also required to complete an academic (theoretical knowledge) qualification, which is delivered either as study days away from the workplace or distance learning. The qualification standards set by the GB pharmacy regulator are currently the Level 3 Diploma in Pharmacy Service Skills (work-based) and Level 3 Diploma in Pharmaceutical Science (theory) [24] which predate mandatory registration of 2011. These standards are still in use up until August 2020.

As the pharmacy technician role has developed, the underpinning education model in GB has failed to keep up with the practice, which has caused considerable confusion for pharmacists and employers as pharmacy technician roles, boundaries, and accountability have been difficult to define [25,26].

Role definition is also a challenge across the globe as both the EAPT survey 7 and the International Pharmaceutical Federation (FIP) 2017 [8] introductory global descriptive survey blend the pharmacy technician role with that of ‘pharmacy support workforce cadres’ who work with pharmacists. Vast global variation compounds the barriers to understanding the pharmacy technician role, the education and competencies needed to underpin their work, and ultimately the autotomy of their professional practice.

Across the USA and Australia, current rules and regulations concerning the education and training of pharmacy technicians varies from state to state, and applying a national certified educational programme is a source of much debate. In comparison however, mirroring GB, Canada’s national model for the regulation of pharmacy technicians exists across 90% of its provinces, and the pharmacy technician title is restricted to those who meet the qualification requirements and are registered with their provincial regulatory body [27].

Acknowledging the changing healthcare landscape and the need to upskill the pharmacy technician workforce, in October 2017, the GB pharmacy regulator published the new Initial Education and Training Standards (IETS) [28]. The outcome-based standards now being introduced (2020) incorporate the shift in knowledge and skills required for patient/person-centred practice. This involves four domains of study: 1. Person-centred care; 2. Professionalism; 3. Professional knowledge and skills; 4. Collaboration. Using Miller’s Pyramid (1990) [29] theory of assessment and competence, the new standards will require building from fundamental knowledge level of ‘knows’, to the application level of ‘knows how’, to measuring competence at the ‘does’ level and having to achieve and exceed at the lower level before moving on.

**Figure 1:**
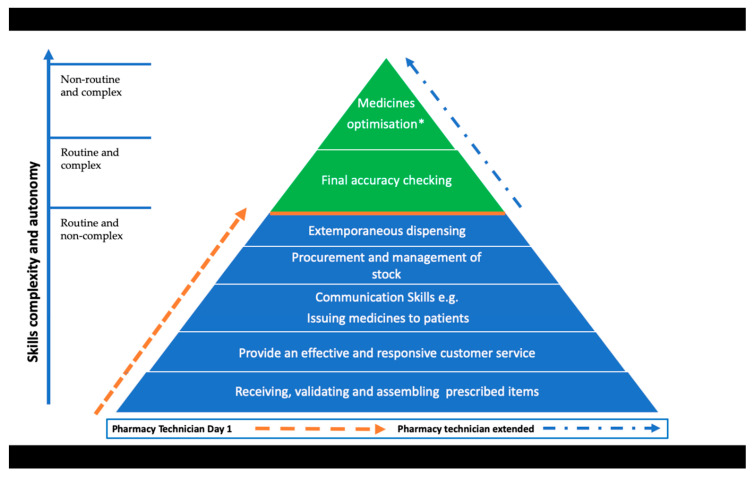
Great Britain (GB)-registered pharmacy technician role and skills—2010 General Pharmaceutical Council (GPhC) education standards.

**Figure 2:**
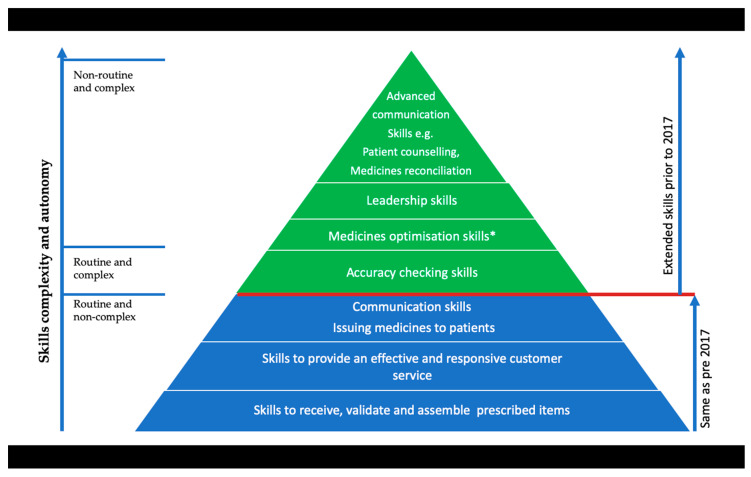
GB-registered pharmacy technician role and skills—2017 GPhC standards.

Whilst providing a broad base of knowledge and skills for work in a range of pharmacy settings across GB, the IETS have a strong emphasis on effective communication to support the clinical, operational, and scientific practices, procedures, and professionalism required of the registered pharmacy technician. Requiring a qualification of a minimum Level 3 (broadly equating to the UK subject advanced level qualifications—A level), Figure 1 and Figure 2 illustrate the comparison of activities from the previous qualification to the new IETS and measures these along a continuum of pharmacy skills. 

Figure 1 illustrates the mandatory skills required for the 2010 GB qualification at day one of practice and extended skills along a continuum of skill complexity and autonomy. It shows inclusion of the traditional skills of dispensing, receiving prescriptions, and managing the stock of the pharmacy. Another inclusion is extemporaneous dispensing skills, sometimes referred to as compounding. In the UK, this skill has become all but non-existent with community pharmacies in particular due to the high risk involved in the preparation, with pharmacies opting to outsource these requests to organisations specifically set up for this type of production. The communication skills required for these standards are at a basic or routine level for handing out prescriptions, giving mainly noncomplex instructions and dealing with routine queries and customer service issues. Figure 1 also illustrates the addition of final accuracy checking. This was the first extended skill to be undertaken by pharmacy technicians approximately 20 years ago, originally in hospital pharmacy but now also widely practiced in community pharmacy. Medicines reconciliation (an activity that is integral to medicines optimisation*) was the second extended role introduced in acute hospitals following the publication of guidance by the National Institute for Health and Care Excellence in 2007 [30] (then called the National Institute for Clinical Excellence). Originally a role for pharmacists, this became a delegated activity and pharmacy technicians have been training to undertake medicines reconciliation over the last 10 years. This activity has increased and is now accepted as part of the pharmacy technicians’ progression and practiced across all settings where there is patients’ transfer of care.

In comparison, the first three levels of Figure 2 show that the core skill of dispensing—in its broadest definition of receiving prescriptions, validating, assembling medicines, and issuing medicine to a named person—remains. The next four levels then illustrate the mandatory activities newly introduced in the 2017 IETs, with extemporaneous dispensing removed from the standards completely as it was considered an obsolete activity. In addition to Medicines Optimisation* and Accuracy Checking, previously widely recognised as extended, advanced communication and leadership skills have been introduced. These changes and additions reflect the expectations of pharmacy technician practice by the GB regulator as well as working within the regulatory Standards for Pharmacy Professionals [1]. The mandatory inclusion of medicines optimisation* and accuracy checking activities which are delivered through person-centred care, commands taking responsibility for the legal, safe, and efficient supply of medicines together with using professional judgement and strategies for continuous quality improvement. The additional skills required to support this competence are now intrinsically embedded within new IETS [26] learning outcomes along with the required leadership and advanced communication skills. Although skills such as procurement and stock management have been removed from the IETS at the ‘does’ level, they have not been removed entirely and PTPTs will need to learn and be assessed at the ‘knows how’ level of the Miller’s pyramid. 

Recognising that the regulatory purpose of the IETS [28] is to ensure that newly registered pharmacy technicians are competent to practise safely and effectively, it is important to acknowledge that competence is much broader than skills alone. Whereas skills are specific to a task or activity, to perform these competently to an acceptable level of ability requires the appropriate depth and breadth of underpinning knowledge and understanding. Thus, professional pharmacy technicians need to be able to identify the required knowledge that underpins their job role and must be able to apply it in practice.

Given that competence is said to build on a foundation of clinical skills, scientific knowledge, and professional development, it also begs the question as to what extent of knowledge is required to combine both the knowledge and competency elements of the evolving job role.

Accepting that Miller’s Pyramid requires achieving and exceeding the preceding level [29], and the regulators expectation that the newly registered pharmacy technician will be acting autonomously and consistently in complex situations [27], albeit defined situations, it can be argued that the level of knowledge required is considerable.

As the GB pharmacy regulatory Professional Standards [1] are very much reflected in the new IETs [28] and these include the complexity of ethical and effective decision making, identifying and responding to errors, and raising concerns, this arguably raises the level and expectations from day 1 of practice and that of a qualification fit for purpose.

## 4. Conclusions

Responding to the pressures of the NHS in delivering patient care, every healthcare profession in the UK has been called upon to maximise their outputs in the most efficient and cost-effective way. The expectations of UK healthcare policy and the integrated care model are already being transposed into new pharmacy services and managing skill mix efficiency will be a major contribution to its success. 

The introduction of a new ‘NHS Discharge Medicines Service’ [30] in addition to the ‘NHS Community Pharmacist Consultation Service’ (already part of the Community Pharmacy Contractual Framework [31]) adds to the expanse of the pharmaceutical care provision, providing development opportunities for community pharmacy technicians. A pharmacy technician qualifying with the new IETs should have the fundamental education and competence to support pharmacists in these services and allow scope for ongoing development. However, the legacy workforce would need additional training to ensure they did not present any risk to patient safety. With this in mind, alongside the expanding role of primary care and news that pharmacy technicians have been added to the list of healthcare professions in the evolving NHSE primary care network structure, undeniably, the primary care pharmacy technician role will also continue to evolve. Thus, there is clearly a significant correlation between progression of pharmacy services, workforce capacity, and utilising pharmacy technicians aligned with their professional knowledge and skills.

There is no doubt that the intention of the new IETS is to embed what was previously seen as extended roles for GB pharmacy technicians into standard practice and develop the profession further. This is indeed required to empower both pharmacists and pharmacy technicians to deliver the aspirations of the NHS England’s Long-term plan [31]. Delivering person-centred care to help patients optimise their medicines and support shared decision-making depends on skilful and proficient communication and skills. Of course, the content of the new qualification must be sound to transfer the acquired behaviours, knowledge, and skills into professional practice. Only time will tell whether the minimum level 3 adequately supports this new, complex role and if two years is sufficient for the trainee to achieve the outcomes and full potential as a healthcare professional. An in-depth evaluation will need to be undertaken once the first trainees come through to explore these aspects.

Moving forward, with the paucity of literature available, larger scale research would provide further insight to reassure the pharmacy and wider healthcare professions, and the public, that the evolving pharmacy technician role presents no increased risk to patient safety and contributes significantly to releasing pharmacists’ time for person-centred clinical activities.

## Figures and Tables

**Table 1:** Medicines optimisation definition and activities undertaken by pharmacy technicians.

*Medicines Optimisation
**Definition**
Medicines optimisation is an approach that seeks to maximise the beneficial clinical outcomes for patients from medicines with an emphasis on safety, governance, professional collaboration, and patient engagement [9].
**Pharmacy Technician activities supporting patient safety include the following:** Communicating with patients, patients’ representatives, and the public providing advice about their medicines. Shared decision making with patients about taking their medicines. Providing patients with compliance aids when required and demonstrating their use. Supplying medicines for individual patients. Assessing appropriateness of medicine forms for patients. Referring complex clinical inventions to pharmacist or prescriber. Providing advice about repeat supplies and storage of medicines. Analysing quantities of medicines to reduce waste and safe disposal. Assessing patients’ own medicines for use ensuring they are fit for purpose. Taking a history of a patient’s medication use. Reconciling a patient’s medicines from one setting to another. Communication with the multidisciplinary team to streamline patient care.
